# Recurrent TET2 mutations in adult T cell leukemia (ATL) and identification of a Single Nucleotide polymorphism in TET2 region predisposing to ATL development

**DOI:** 10.1186/1742-4690-12-S1-O12

**Published:** 2015-08-28

**Authors:** Ambroise Marçais, Katia Hanssens, Laetitia Waast, Vahid Asnafi, Patrice Dubreuil, Antoine Gessain, Claudine Pique, Olivier Hermine

**Affiliations:** 1 Service d'hématologie, hôpital Universitaire Necker, Université René Descartes, Institut Imagine, Inserm, U1016, Institut Cochin, Paris, France; 2 Inserm U1068, Centre de Recherche en Cancérologie de Marseille, Institut Paoli-Calmettes, Université de la Méditerranée, Marseille, France; 3 Inserm, U1016, Institut Cochin, Paris, France; 4 Department d'hématologie, hôpital universitaire Necker, Université René Descartes, Paris, France; 5 Unité d'épidémiologie et Physiopathologie des Virus Oncogènes, Département de Virologie, CNRS, UMR 3569, Institut Pasteur, Paris, France; 6 Service d'hématologie, Hôpital Universitaire Necker, Université René Descartes, Institut Imagine, Inserm, Paris, France

## 

Deregulation of DNA methylation, such as inactivation of the Ten-Eleven Translocation 2 (TET2) gene by haplo-insufficiency, has been recently identified in malignant hematologic diseases. Inactivating mutations of TET2 were first described in myeloid disorders and more recently in peripheral T-cell lymphomas especially those that are harboring T follicular helper features like angio-immunoblastic T cell lymphoma. In order to determine new oncogenic pathways in Adult T cell leukemia/lymphoma (ATLL), we investigated the presence of TET2 coding sequence mutations and their clinical relevance in a retrospective cohort of 66 ATL patients. We identified mono allelic inactivating mutations of TET2 gene in 12 patients of 66 analyzed (18%). Of the 51 patients with aggressive forms (acute and lymphoma), 11 (22%) had TET2 mutations while only one (7%) of the 12 patients with indolent forms had a TET2 mutation. Of the 12 mutated patients, 8 showed the same recurrent point mutation known as a Single Nucleotide polymorphism (SNP), which creates a frameshift resulting in the introduction of premature stop codon in one allele and lead to haplo-insufficiency. We have characterized this SNP and demonstrated that the mutated gene encodes for a truncated form of Tet2 that is no longer catalytically active. We then addressed whether this mutation could predispose to ATL development by sequencing the TET2 SNP region in 50 HTLV-1 carriers from French Guyana matching the African or French Caribbean origins of the patients. We found that the percentage of the mutation in this control population was around 4%, which is similar to that of the African population and is three fold less than in our ATL cohort. This finding suggests that this mutation could be a predisposal factor for developing an ATL. In conclusion, we have showed that TET2 mutations are frequently associated with ATL, notably the aggressive forms, and that a TET2 SNP may predispose to ATL development.

